# (*E*)-3-(2,4-Dimeth­oxy­phen­yl)-1-(3,4,5-trimeth­oxy­phen­yl)prop-2-en-1-one

**DOI:** 10.1107/S1600536811053001

**Published:** 2011-12-17

**Authors:** Jianzhang Wu, Junyan Qiu, Xiaokai Wu, Shulin Yang, Yonggen Liu

**Affiliations:** a Institute of Biotechnology, Nanjing University of Science and Technology, Nanjing, Jiangsu Province 210094, People’s Republic of China; b School of Pharmacy, Wenzhou Medical College, Wenzhou, Zhejiang Province 325035, People’s Republic of China; c Life Science College, Wenzhou Medical College, Wenzhou, Zhejiang Province 325035, People’s Republic of China; d School of Pharmacy, Hainan Medical College, Haikou, Hainan Province 571101, People’s Republic of China

## Abstract

In the title chalcone derivative, C_20_H_22_O_6_, the dihedral angle between the mean planes of the benzene rings is 15.77 (6)°. The H atoms of the central C=C double bond are in a *trans* configuration. There are a number of C—H⋯O interactions and a C—H⋯π interaction present in the crystal structure.

## Related literature

For related structures, see: Wu *et al.* (2010 ▶, 2011 ▶); Huang *et al.* (2010 ▶); Peng *et al.* (2010 ▶). For applications of chalcones, see: Wu *et al.* (2010 ▶, 2011 ▶); Nielsen *et al.* (2005 ▶). For the hydrogen-bond analysis, see: Spek (2009 ▶).
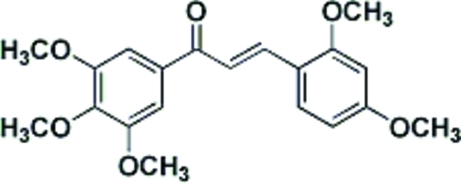


## Experimental

### 

#### Crystal data

C_20_H_22_O_6_
*M* *_r_* = 358.38
Monoclinic, 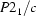

*a* = 8.3111 (11) Å
*b* = 13.8493 (17) Å
*c* = 15.887 (2) Å
β = 101.588 (2)°
*V* = 1791.4 (4) Å^3^
*Z* = 4
Mo *K*α radiation
μ = 0.10 mm^−1^
*T* = 293 K
0.40 × 0.37 × 0.31 mm

#### Data collection

Bruker SMART CCD diffractometer
Absorption correction: multi-scan (*SADABS*; Bruker, 2001 ▶) *T*_min_ = 0.651, *T*_max_ = 1.000
9663 measured reflections
3512 independent reflections
2452 reflections with *I* > 2σ(*I*)
*R*_int_ = 0.045

#### Refinement

*R*[*F* ^2^ > 2σ(*F* ^2^)] = 0.046
*wR*(*F* ^2^) = 0.126
*S* = 0.97
3512 reflections
241 parameters
H-atom parameters constrained
Δρ_max_ = 0.20 e Å^−3^
Δρ_min_ = −0.19 e Å^−3^

### 

Data collection: *SMART* (Bruker, 2001 ▶); cell refinement: *SAINT* (Bruker, 2001 ▶); data reduction: *SAINT*; program(s) used to solve structure: *SHELXS97* (Sheldrick, 2008 ▶); program(s) used to refine structure: *SHELXL97* (Sheldrick, 2008 ▶); molecular graphics: *SHELXTL* (Sheldrick, 2008 ▶); software used to prepare material for publication: *SHELXTL*.

## Supplementary Material

Crystal structure: contains datablock(s) I, global. DOI: 10.1107/S1600536811053001/zq2146sup1.cif

Structure factors: contains datablock(s) I. DOI: 10.1107/S1600536811053001/zq2146Isup2.hkl

Supplementary material file. DOI: 10.1107/S1600536811053001/zq2146Isup3.cml

Additional supplementary materials:  crystallographic information; 3D view; checkCIF report

## Figures and Tables

**Table 1:** Hydrogen-bond geometry (Å, °) *Cg*1 is the centroid of the C4–C9 ring.

*D*—H⋯*A*	*D*—H	H⋯*A*	*D*⋯*A*	*D*—H⋯*A*
C8—H8⋯O6^i^	0.93	2.65	3.562 (2)	166
C18—H18*B*⋯O3^ii^	0.96	2.64	3.404 (2)	136
C16—H16*B*⋯O2^iii^	0.96	2.68	3.389 (2)	132
C16—H16*C*⋯O1^iii^	0.96	2.66	3.608 (3)	169
C19—H19*C*⋯*Cg*1^iv^	0.96	2.79	3.530 (2)	134

Symmetry codes: (i) 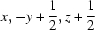; (ii) 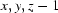; (iii) 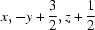; (iv) 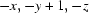.

**Fig. 1.**
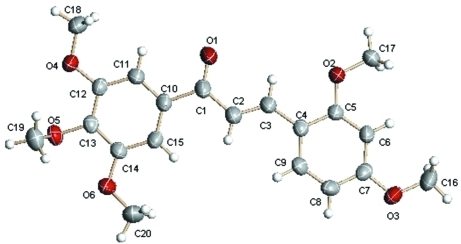
Molecular structure of the title compound. Displacement ellipsoids are drawn at the 30% probability level.

**Fig. 2.**
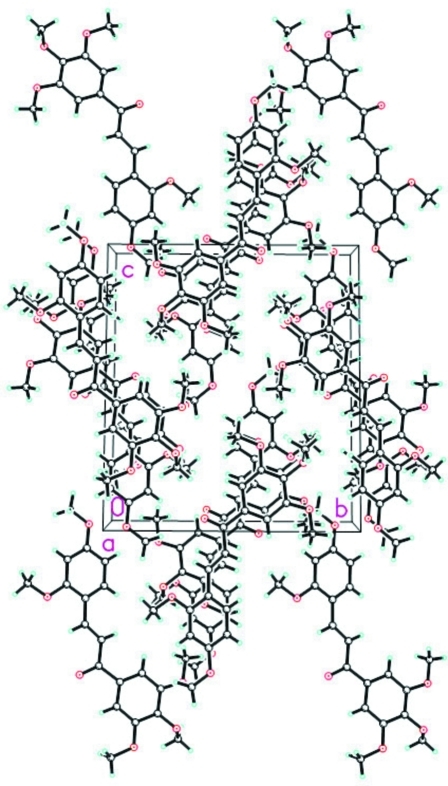
Crystal packing of the title compound viewed down the a axis.

## supplementary crystallographic information

### Comment

Chalcones are characterized by possessing two aromatic rings linked by a three-carbon α,β-unsaturated carbonyl system (Wu *et al.*, 2010; Wu *et al.*, 2011). Natural chalcones have many kinds of active biological properties such as antiinflammatory, antitumoral, antimalarial, antileishmanial, Antibacterial. Investigations have demonstrated that synthetical chalcones have the same biological properties as natural chalcones. (Wu *et al.* 2010; Wu *et al.* 2011; Nielsen *et al.* 2005). In order to study its anticancer agents, we have synthesized the title chalcone derivative and herein its crystal structure is reported. The crystal structure parameters are similar to those found in some analogous structures reported in the literature (Peng *et al.*, 2010; Huang *et al.*, 2010).The dihedral angle between the mean planes of the phenyl rings is 15.77 (6)°. The H atoms of the central C=C double bond are in a *trans* configuration. In the crystal structure, there are many weak C–H···O intermolecular contacts (Table 1) but no classic hydrogen bonds between the molecules (Spek, 2009).

### Experimental

The title compound was synthesized by Claisen-Schmidt condensation between 2,4-dimethoxybenzaldehyde and 1-(3,4,5-trimethoxyphenyl)ethanone. 2,4-Dimethoxybenzaldehyde (1 mmol) and 1-(3,4,5-trimethoxyphenyl)ethanone (1 mmol) were dissolved in ehanol (20 ml). The reaction temperature was controlled at 283 K, and then NaOH (20%, 3 drops) was added. The reaction was monitored by thin-layer chromatography. 20 ml H_2_O was added 5 h later and the yellow solid precipitated. It was washed with a mixture of water and cold ethanol, and dried (yield: 70%; mp 125.7–127.7°C). Single crystals of the title compound were obtained by recrystallization from a solution of CH_3_CH_2_OH / CH_2_Cl_2_ at 293 K.

### Refinement

All H atoms were placed in geometrical positions and constrained to ride on their parent atoms, with C—H = 0.93 Å and *U*_iso_(H) = 1.2*U*_eq_(C) for aromatic H atoms, and with C—H = 0.96 Å and *U*_iso_(H) = 1.5*U*_eq_(C) for methyl H atoms.

### Crystal data

**Table:**

C_20_H_22_O_6_	*F*(000) = 760
*M**_r_* = 358.38	*D*_x_ = 1.329 Mg m^−^^3^
Monoclinic, *P*2_1_/*c*	Mo *K*α radiation, λ = 0.71073 Å
Hall symbol: -P 2ybc	Cell parameters from 2728 reflections
*a* = 8.3111 (11) Å	θ = 5.2–49.2°
*b* = 13.8493 (17) Å	µ = 0.10 mm^−^^1^
*c* = 15.887 (2) Å	*T* = 293 K
β = 101.588 (2)°	Prismatic, green
*V* = 1791.4 (4) Å^3^	0.40 × 0.37 × 0.31 mm
*Z* = 4	

### Data collection

**Table:**

Bruker SMART CCD diffractometer	3512 independent reflections
Radiation source: fine-focus sealed tube	2452 reflections with *I* > 2σ(*I*)
graphite	*R*_int_ = 0.045
phi and ω scans	θ_max_ = 26.0°, θ_min_ = 2.0°
Absorption correction: multi-scan (*SADABS*; Bruker, 2001)	*h* = −10→9
*T*_min_ = 0.651, *T*_max_ = 1.000	*k* = −17→15
9663 measured reflections	*l* = −16→19

### Refinement

**Table:**

Refinement on *F*^2^	Secondary atom site location: difference Fourier map
Least-squares matrix: full	Hydrogen site location: inferred from neighbouring sites
*R*[*F*^2^ > 2σ(*F*^2^)] = 0.046	H-atom parameters constrained
*wR*(*F*^2^) = 0.126	*w* = 1/[σ^2^(*F*_o_^2^) + (0.0689*P*)^2^] where *P* = (*F*_o_^2^ + 2*F*_c_^2^)/3
*S* = 0.97	(Δ/σ)_max_ < 0.001
3512 reflections	Δρ_max_ = 0.20 e Å^−^^3^
241 parameters	Δρ_min_ = −0.19 e Å^−^^3^
0 restraints	Extinction correction: *SHELXL97* (Sheldrick, 2008), Fc^*^=kFc[1+0.001xFc^2^λ^3^/sin(2θ)]^-1/4^
Primary atom site location: structure-invariant direct methods	Extinction coefficient: 0.0069 (15)

### Special details

**Table:**

Geometry. All e.s.d.'s (except the e.s.d. in the dihedral angle between two l.s. planes) are estimated using the full covariance matrix. The cell e.s.d.'s are taken into account individually in the estimation of e.s.d.'s in distances, angles and torsion angles; correlations between e.s.d.'s in cell parameters are only used when they are defined by crystal symmetry. An approximate (isotropic) treatment of cell e.s.d.'s is used for estimating e.s.d.'s involving l.s. planes.
Refinement. Refinement of *F*^2^ against ALL reflections. The weighted *R*-factor *wR* and goodness of fit *S* are based on *F*^2^, conventional *R*-factors *R* are based on *F*, with *F* set to zero for negative *F*^2^. The threshold expression of *F*^2^ > σ(*F*^2^) is used only for calculating *R*-factors(gt) *etc*. and is not relevant to the choice of reflections for refinement. *R*-factors based on *F*^2^ are statistically about twice as large as those based on *F*, and *R*- factors based on ALL data will be even larger.

### Fractional atomic coordinates and isotropic or equivalent isotropic displacement parameters (Å^2^)

**Table:**

	*x*	*y*	*z*	*U*_iso_*/*U*_eq_	
O1	0.3078 (3)	0.60842 (10)	−0.03257 (9)	0.0969 (7)	
O2	0.45908 (16)	0.73756 (8)	0.24726 (7)	0.0510 (4)	
O3	0.34229 (18)	0.58134 (9)	0.49898 (7)	0.0629 (4)	
O4	0.20538 (17)	0.38935 (8)	−0.28996 (7)	0.0549 (4)	
O5	0.11723 (15)	0.21987 (8)	−0.23292 (7)	0.0487 (3)	
O6	0.07241 (18)	0.19802 (8)	−0.07336 (8)	0.0614 (4)	
C1	0.2622 (3)	0.53394 (12)	−0.00427 (11)	0.0532 (5)	
C2	0.2513 (2)	0.52558 (12)	0.08616 (11)	0.0494 (5)	
H2	0.1994	0.4719	0.1037	0.059*	
C3	0.3124 (2)	0.59147 (12)	0.14391 (11)	0.0478 (5)	
H3	0.3597	0.6450	0.1231	0.057*	
C4	0.3155 (2)	0.59116 (11)	0.23532 (10)	0.0397 (4)	
C5	0.3969 (2)	0.66447 (11)	0.28813 (10)	0.0395 (4)	
C6	0.4097 (2)	0.66259 (11)	0.37629 (10)	0.0438 (4)	
H6	0.4670	0.7109	0.4104	0.053*	
C7	0.3368 (2)	0.58853 (12)	0.41331 (11)	0.0454 (4)	
C8	0.2505 (2)	0.51675 (12)	0.36245 (12)	0.0495 (5)	
H8	0.1993	0.4677	0.3872	0.059*	
C9	0.2416 (2)	0.51894 (11)	0.27580 (11)	0.0456 (5)	
H9	0.1839	0.4703	0.2423	0.055*	
C10	0.2178 (2)	0.44929 (11)	−0.06312 (10)	0.0441 (5)	
C11	0.2324 (2)	0.46042 (11)	−0.14787 (11)	0.0451 (4)	
H11	0.2675	0.5192	−0.1662	0.054*	
C12	0.1956 (2)	0.38529 (11)	−0.20524 (10)	0.0428 (4)	
C13	0.1453 (2)	0.29723 (11)	−0.17775 (10)	0.0407 (4)	
C14	0.1279 (2)	0.28626 (11)	−0.09326 (11)	0.0436 (4)	
C15	0.1647 (2)	0.36210 (11)	−0.03595 (11)	0.0468 (5)	
H15	0.1538	0.3545	0.0208	0.056*	
C16	0.4271 (3)	0.65505 (15)	0.55295 (12)	0.0817 (8)	
H16A	0.5415	0.6534	0.5505	0.123*	
H16B	0.4147	0.6445	0.6110	0.123*	
H16C	0.3821	0.7169	0.5336	0.123*	
C17	0.5458 (2)	0.81254 (12)	0.29860 (11)	0.0517 (5)	
H17A	0.4751	0.8421	0.3321	0.078*	
H17B	0.5805	0.8601	0.2621	0.078*	
H17C	0.6402	0.7859	0.3363	0.078*	
C18	0.2455 (3)	0.47971 (13)	−0.32250 (12)	0.0632 (6)	
H18A	0.1629	0.5263	−0.3168	0.095*	
H18B	0.2508	0.4730	−0.3820	0.095*	
H18C	0.3501	0.5010	−0.2907	0.095*	
C19	−0.0442 (2)	0.21605 (14)	−0.28238 (13)	0.0635 (6)	
H19A	−0.1204	0.2052	−0.2452	0.095*	
H19B	−0.0523	0.1643	−0.3232	0.095*	
H19C	−0.0697	0.2761	−0.3123	0.095*	
C20	0.0313 (3)	0.18677 (15)	0.00746 (13)	0.0796 (7)	
H20A	0.1280	0.1943	0.0515	0.119*	
H20B	−0.0141	0.1236	0.0115	0.119*	
H20C	−0.0483	0.2347	0.0147	0.119*	

### Atomic displacement parameters (Å^2^)

**Table:**

	*U*^11^	*U*^22^	*U*^33^	*U*^12^	*U*^13^	*U*^23^
O1	0.196 (2)	0.0472 (8)	0.0459 (9)	−0.0398 (10)	0.0199 (10)	−0.0057 (6)
O2	0.0720 (9)	0.0430 (7)	0.0373 (7)	−0.0161 (6)	0.0091 (6)	−0.0008 (5)
O3	0.1002 (12)	0.0532 (8)	0.0383 (7)	−0.0019 (7)	0.0214 (7)	0.0041 (6)
O4	0.0851 (10)	0.0457 (7)	0.0366 (7)	−0.0065 (6)	0.0188 (6)	−0.0026 (5)
O5	0.0564 (8)	0.0391 (6)	0.0498 (7)	0.0031 (6)	0.0088 (6)	−0.0121 (5)
O6	0.0974 (11)	0.0389 (7)	0.0520 (8)	−0.0099 (7)	0.0252 (7)	−0.0010 (6)
C1	0.0786 (14)	0.0389 (10)	0.0384 (10)	−0.0011 (9)	0.0028 (9)	−0.0016 (8)
C2	0.0649 (13)	0.0396 (9)	0.0429 (10)	−0.0037 (9)	0.0092 (9)	−0.0034 (8)
C3	0.0658 (13)	0.0363 (9)	0.0400 (10)	−0.0048 (8)	0.0076 (8)	−0.0006 (7)
C4	0.0486 (11)	0.0334 (8)	0.0364 (9)	0.0030 (7)	0.0067 (8)	−0.0017 (7)
C5	0.0473 (10)	0.0338 (8)	0.0375 (9)	−0.0002 (7)	0.0087 (8)	0.0010 (7)
C6	0.0574 (12)	0.0376 (9)	0.0353 (9)	−0.0013 (8)	0.0065 (8)	−0.0034 (7)
C7	0.0625 (12)	0.0395 (9)	0.0361 (10)	0.0095 (8)	0.0141 (8)	0.0039 (7)
C8	0.0659 (13)	0.0354 (9)	0.0514 (11)	−0.0025 (8)	0.0217 (9)	0.0041 (8)
C9	0.0551 (12)	0.0338 (9)	0.0484 (11)	−0.0019 (8)	0.0119 (9)	−0.0064 (7)
C10	0.0560 (12)	0.0377 (9)	0.0353 (9)	0.0018 (8)	0.0018 (8)	−0.0013 (7)
C11	0.0581 (12)	0.0353 (9)	0.0410 (10)	−0.0034 (8)	0.0078 (8)	0.0011 (7)
C12	0.0520 (11)	0.0412 (9)	0.0354 (9)	0.0038 (8)	0.0094 (8)	−0.0016 (7)
C13	0.0466 (11)	0.0343 (9)	0.0402 (10)	0.0047 (7)	0.0066 (8)	−0.0052 (7)
C14	0.0537 (11)	0.0328 (9)	0.0440 (10)	0.0007 (8)	0.0086 (8)	0.0008 (7)
C15	0.0641 (13)	0.0422 (10)	0.0334 (9)	0.0020 (9)	0.0082 (8)	0.0010 (7)
C16	0.138 (2)	0.0694 (14)	0.0348 (11)	−0.0064 (14)	0.0103 (12)	−0.0023 (10)
C17	0.0619 (13)	0.0421 (10)	0.0515 (11)	−0.0131 (9)	0.0125 (9)	−0.0037 (8)
C18	0.0934 (17)	0.0537 (12)	0.0456 (11)	−0.0055 (11)	0.0211 (11)	0.0058 (9)
C19	0.0649 (14)	0.0589 (12)	0.0627 (13)	−0.0058 (10)	0.0032 (10)	−0.0159 (10)
C20	0.117 (2)	0.0644 (14)	0.0642 (14)	−0.0289 (14)	0.0338 (13)	0.0030 (11)

### Geometric parameters (Å, °)

**Table:**

O1—C1	1.216 (2)	C9—H9	0.9300
O2—C5	1.3595 (19)	C10—C15	1.384 (2)
O2—C17	1.4235 (18)	C10—C11	1.385 (2)
O3—C7	1.3564 (19)	C11—C12	1.377 (2)
O3—C16	1.425 (2)	C11—H11	0.9300
O4—C12	1.366 (2)	C12—C13	1.388 (2)
O4—C18	1.419 (2)	C13—C14	1.387 (2)
O5—C13	1.3742 (18)	C14—C15	1.383 (2)
O5—C19	1.413 (2)	C15—H15	0.9300
O6—C14	1.3656 (19)	C16—H16A	0.9600
O6—C20	1.402 (2)	C16—H16B	0.9600
C1—C2	1.462 (2)	C16—H16C	0.9600
C1—C10	1.499 (2)	C17—H17A	0.9600
C2—C3	1.321 (2)	C17—H17B	0.9600
C2—H2	0.9300	C17—H17C	0.9600
C3—C4	1.447 (2)	C18—H18A	0.9600
C3—H3	0.9300	C18—H18B	0.9600
C4—C9	1.396 (2)	C18—H18C	0.9600
C4—C5	1.402 (2)	C19—H19A	0.9600
C5—C6	1.383 (2)	C19—H19B	0.9600
C6—C7	1.381 (2)	C19—H19C	0.9600
C6—H6	0.9300	C20—H20A	0.9600
C7—C8	1.387 (2)	C20—H20B	0.9600
C8—C9	1.364 (2)	C20—H20C	0.9600
C8—H8	0.9300		
			
C5—O2—C17	117.82 (13)	C11—C12—C13	119.79 (16)
C7—O3—C16	117.64 (15)	O5—C13—C14	119.64 (14)
C12—O4—C18	117.40 (13)	O5—C13—C12	120.52 (15)
C13—O5—C19	113.86 (13)	C14—C13—C12	119.82 (14)
C14—O6—C20	117.86 (14)	O6—C14—C15	124.57 (16)
O1—C1—C2	121.06 (15)	O6—C14—C13	115.33 (14)
O1—C1—C10	119.26 (16)	C15—C14—C13	120.10 (15)
C2—C1—C10	119.67 (15)	C14—C15—C10	119.96 (16)
C3—C2—C1	122.45 (17)	C14—C15—H15	120.0
C3—C2—H2	118.8	C10—C15—H15	120.0
C1—C2—H2	118.8	O3—C16—H16A	109.5
C2—C3—C4	128.42 (17)	O3—C16—H16B	109.5
C2—C3—H3	115.8	H16A—C16—H16B	109.5
C4—C3—H3	115.8	O3—C16—H16C	109.5
C9—C4—C5	116.56 (15)	H16A—C16—H16C	109.5
C9—C4—C3	122.93 (14)	H16B—C16—H16C	109.5
C5—C4—C3	120.50 (15)	O2—C17—H17A	109.5
O2—C5—C6	122.56 (14)	O2—C17—H17B	109.5
O2—C5—C4	115.95 (14)	H17A—C17—H17B	109.5
C6—C5—C4	121.48 (15)	O2—C17—H17C	109.5
C7—C6—C5	119.61 (15)	H17A—C17—H17C	109.5
C7—C6—H6	120.2	H17B—C17—H17C	109.5
C5—C6—H6	120.2	O4—C18—H18A	109.5
O3—C7—C6	123.55 (15)	O4—C18—H18B	109.5
O3—C7—C8	116.18 (16)	H18A—C18—H18B	109.5
C6—C7—C8	120.26 (16)	O4—C18—H18C	109.5
C9—C8—C7	119.28 (16)	H18A—C18—H18C	109.5
C9—C8—H8	120.4	H18B—C18—H18C	109.5
C7—C8—H8	120.4	O5—C19—H19A	109.5
C8—C9—C4	122.75 (16)	O5—C19—H19B	109.5
C8—C9—H9	118.6	H19A—C19—H19B	109.5
C4—C9—H9	118.6	O5—C19—H19C	109.5
C15—C10—C11	119.75 (15)	H19A—C19—H19C	109.5
C15—C10—C1	122.81 (15)	H19B—C19—H19C	109.5
C11—C10—C1	117.44 (15)	O6—C20—H20A	109.5
C12—C11—C10	120.55 (16)	O6—C20—H20B	109.5
C12—C11—H11	119.7	H20A—C20—H20B	109.5
C10—C11—H11	119.7	O6—C20—H20C	109.5
O4—C12—C11	124.89 (16)	H20A—C20—H20C	109.5
O4—C12—C13	115.31 (14)	H20B—C20—H20C	109.5
			
O1—C1—C2—C3	−10.4 (3)	O1—C1—C10—C11	0.4 (3)
C10—C1—C2—C3	169.13 (18)	C2—C1—C10—C11	−179.08 (17)
C1—C2—C3—C4	−177.75 (18)	C15—C10—C11—C12	−0.4 (3)
C2—C3—C4—C9	−3.8 (3)	C1—C10—C11—C12	179.24 (17)
C2—C3—C4—C5	175.28 (19)	C18—O4—C12—C11	−4.6 (3)
C17—O2—C5—C6	2.4 (2)	C18—O4—C12—C13	175.86 (16)
C17—O2—C5—C4	−178.70 (15)	C10—C11—C12—O4	179.65 (16)
C9—C4—C5—O2	−175.91 (14)	C10—C11—C12—C13	−0.8 (3)
C3—C4—C5—O2	5.0 (2)	C19—O5—C13—C14	94.45 (19)
C9—C4—C5—C6	3.0 (2)	C19—O5—C13—C12	−87.4 (2)
C3—C4—C5—C6	−176.14 (16)	O4—C12—C13—O5	3.3 (2)
O2—C5—C6—C7	176.89 (15)	C11—C12—C13—O5	−176.24 (15)
C4—C5—C6—C7	−1.9 (3)	O4—C12—C13—C14	−178.56 (15)
C16—O3—C7—C6	0.5 (3)	C11—C12—C13—C14	1.9 (3)
C16—O3—C7—C8	−178.72 (18)	C20—O6—C14—C15	7.9 (3)
C5—C6—C7—O3	−179.56 (16)	C20—O6—C14—C13	−171.57 (17)
C5—C6—C7—C8	−0.4 (3)	O5—C13—C14—O6	−4.1 (2)
O3—C7—C8—C9	−179.29 (16)	C12—C13—C14—O6	177.80 (15)
C6—C7—C8—C9	1.5 (3)	O5—C13—C14—C15	176.41 (15)
C7—C8—C9—C4	−0.3 (3)	C12—C13—C14—C15	−1.7 (3)
C5—C4—C9—C8	−1.9 (3)	O6—C14—C15—C10	−178.97 (16)
C3—C4—C9—C8	177.22 (17)	C13—C14—C15—C10	0.5 (3)
O1—C1—C10—C15	−180.0 (2)	C11—C10—C15—C14	0.6 (3)
C2—C1—C10—C15	0.6 (3)	C1—C10—C15—C14	−179.05 (17)

### Hydrogen-bond geometry (°)

**Table:**

Cg1 is the centroid of the C4–C9 ring.

**Table:**

*D*—H···*A*
—···
—···
—···
—···
—···
